# The PATHFINDER Study: Assessment of the Implementation of an Investigational Multi-Cancer Early Detection Test into Clinical Practice

**DOI:** 10.3390/cancers13143501

**Published:** 2021-07-13

**Authors:** Lincoln D. Nadauld, Charles H. McDonnell, Tomasz M. Beer, Minetta C. Liu, Eric A. Klein, Andrew Hudnut, Richard A. Whittington, Bruce Taylor, Geoffrey R. Oxnard, Jafi Lipson, Margarita Lopatin, Rita Shaknovich, Karen C. Chung, Eric T. Fung, Deborah Schrag, Catherine R. Marinac

**Affiliations:** 1 Hematology/Oncology, Intermountain Healthcare, St. George, UT 84790, USA; 2 Sutter Health, Sacramento, CA 95816, USA; McDonnC@sutterhealth.org (C.H.M.III); hudnuta@sutterhealth.org (A.H.); 3 Hematology/Medical Oncology, Oregon Health & Science University Knight Cancer Institute, Portland, OR 97239, USA; beert@ohsu.edu; 4 Departments of Oncology and Laboratory Medicine & Pathology, Mayo Clinic, Rochester, MN 55905, USA; liu.minetta@mayo.edu; 5 Glickman Urological and Kidney Institute, Cleveland Clinic, Cleveland, OH 44195, USA; kleine@ccf.org; 6 Department of Internal Medicine, Intermountain Healthcare, Salt Lake City, UT 84111, USA; richard.whittington@imail.org (R.A.W.); bruce.taylor@imail.org (B.T.); 7 Department of Medical Oncology, Division of Population Sciences, Dana-Farber Cancer Institute, Boston, MA 02215, USA; goxnard@foundationmedicine.com (G.R.O.); Deb_Schrag@dfci.harvard.edu (D.S.); CatherineR_Marinac@DFCI.HARVARD.EDU (C.R.M.); 8 Radiology Department, Stanford Hospital and Clinics, Stanford, CA 94305, USA; jlipson@stanford.edu; 9 GRAIL, Inc., Menlo Park, CA 94025, USA; rlopatin@grailbio.com (M.L.); rshaknovich@grailbio.com (R.S.); kchung@grailbio.com (K.C.C.); efung@grailbio.com (E.T.F.)

**Keywords:** methylation cell-free DNA, multi-cancer early detection test, cancer, diagnostic pathways

## Abstract

**Simple Summary:**

PATHFINDER is an interventional study that will examine how well a multi-cancer early detection test can be integrated into clinical practice. This test looks at DNA methylation patterns in patient blood samples to detect cancer and also predict cancer origin. The PATHFINDER study will include ~6200 study participants from 31 sites in the United States. The study will return tests results to participants and their health care providers and will evaluate how test results affect the clinical pathway to confirm or rule out a cancer diagnosis. Results of this study could help determine how a blood-based multi-cancer early detection test will fit into clinical practice.

**Abstract:**

To examine the extent of the evaluation required to achieve diagnostic resolution and the test performance characteristics of a targeted methylation cell-free DNA (cfDNA)-based multi-cancer early detection (MCED) test, ~6200 participants ≥50 years with (cohort A) or without (cohort B) ≥1 of 3 additional specific cancer risk factors will be enrolled in PATHFINDER (NCT04241796), a prospective, longitudinal, interventional, multi-center study. Plasma cfDNA from blood samples will be analyzed to detect abnormally methylated DNA associated with cancer (i.e., cancer “signal”) and a cancer signal origin (i.e., tissue of origin). Participants with a “signal detected” will undergo further diagnostic evaluation per guiding physician discretion; those with a “signal not detected” will be advised to continue guideline-recommended screening. The primary objective will be to assess the number and types of subsequent diagnostic tests needed for diagnostic resolution. Based on microsimulations (using estimates of cancer incidence and dwell times) of the typical risk profiles of anticipated participants, the median (95% CI) number of participants with a “signal detected” result is expected to be 106 (87–128). Subsequent diagnostic evaluation is expected to detect 52 (39–67) cancers. The positive predictive value of the MCED test is expected to be 49% (39–58%). PATHFINDER will evaluate the integration of a cfDNA-based MCED test into existing clinical cancer diagnostic pathways. The study design of PATHFINDER is described here.

## 1. Introduction

Currently, there are a limited number of cancer screening programs in the United States (US), Europe, and other countries. The United States Preventive Services Task Force recommendations are limited to screening for breast, cervical, colon, lung (in high-risk individuals), and, on an individual basis, prostate cancer [1,2,3,4,5]. Similarly, the European Code Against Cancer recommends screening for breast, cervical, and colon cancer [6], while the World Health Organization advocates cervical cancer screening [7]. Despite variable uptake, especially in the underserved [8,9], these programs have decreased cancer mortality [8,10,11,12,13]. Importantly, in the US, approximately three-quarters of all new cancer cases and cancer-related deaths in 2020 will be due to cancers without established screening paradigms [14]. These cancers tend to be diagnosed at later stages [15] when a cure is less likely, and treatment is less effective, more burdensome, and costly [16,17]. Additionally, undergoing multiple single-cancer detection tests results in a cumulative risk of false positives [18].

Many cancers lack screening programs, partly because those cancers have low individual prevalence [19]. A test that screens for multiple cancers simultaneously may overcome this limitation by aggregating prevalence and creating a cost-effective strategy to screen for less common but lethal malignancies. Blood is an attractive analyte for a multi-cancer detection test because almost all cancers pass tumor elements into the circulation [19]. Indeed, several tests analyzing circulating biomarkers are being developed and tested in large, prospective clinical trials [20]. The DETECT-A study (*n =* 10,006) recently demonstrated the feasibility and safety of a multi-analyte (DNA and protein) multi-cancer early detection (MCED) test, CancerSEEK, to detect multiple cancers [21,22]. Using a different approach, a cell-free DNA (cfDNA) methylation-based MCED assay is being developed in one of the largest known clinical genomics programs (*n* > 145,000). This program comprises four clinical studies to develop, validate, and assess the clinical implementation of an MCED test, similar to the approach recommended for cancer screening biomarker development [23]. The foundational Circulating Cell-free Genome Atlas (CCGA) study (NCT02889978) [24] demonstrated that whole-genome bisulfite sequencing outperformed whole-genome sequencing and targeted sequencing analyzing copy-number and single-nucleotide variants, respectively. Further refinement of this approach resulted in a targeted methylation-based MCED test, which analyzes methylation patterns in cell-free DNA shed from tumors to detect signals associated with cancer and has been analytically validated using samples from patients collected at the time of cancer diagnosis before intervention [25]. The assay can detect cancer-specific DNA methylation signatures (“signals”) from >50 cancer types, with a single, fixed false-positive rate of 0.7%, and predicts cancer signal origin (CSO; i.e., tissue of origin) with 93% accuracy (among cancer participants with the signal detected) [24].

Two large, prospective, multi-center, observational cohort studies (STRIVE (NCT03085888) [26] and SUMMIT [NCT03934866] [27]) will be conducted to clinically validate this MCED test in intended-use populations. STRIVE (*n* ≈ 100,000) will enroll women without a cancer diagnosis who are undergoing screening mammography. SUMMIT (*n* ≈ 25,000) will enroll individuals without a cancer diagnosis who are at high risk for lung cancer.

PATHFINDER (NCT04241796), an interventional study that will be the first to return this MCED test’s results to healthcare providers and participants, will evaluate cancer diagnostic and care pathways following a “signal detected” MCED test result. PATHFINDER will also evaluate test performance characteristics and participant-reported outcomes (PROs). Here, we describe the PATHFINDER study design.

## 2. Materials and Methods

### 2.1. Study Design

**Figure 1:**
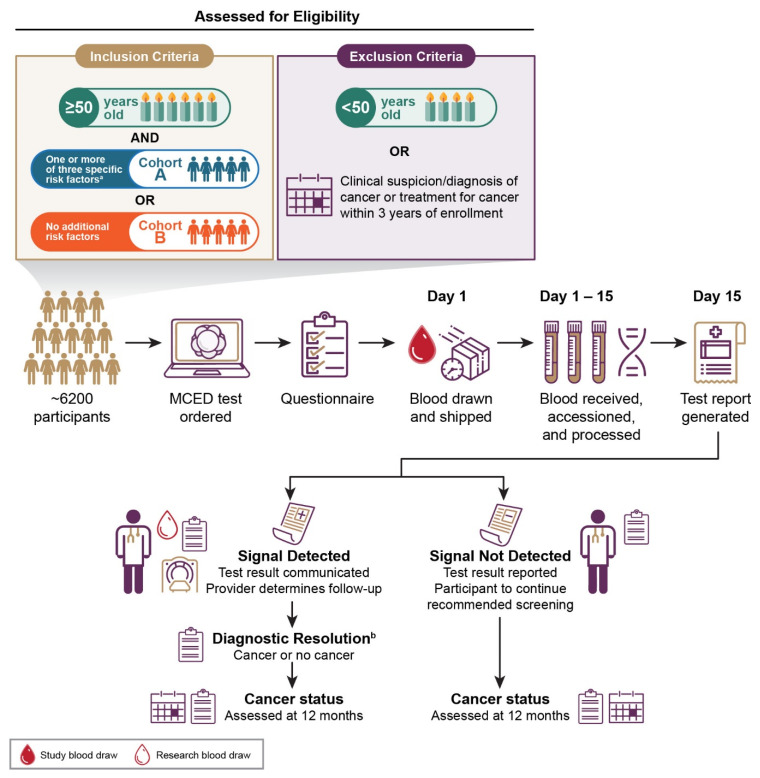
An overview of PATHFINDER study to evaluate the clinical implementation of a cell-free DNA-based targeted methylation multi-cancer early detection (MCED) test. Individuals 50 years or older are eligible to participate and will be divided into 2 cohorts depending on the presence or absence of additional cancer risk factors beyond age. Blood will be collected from ~6200 participants and analyzed with the MCED test. Test results (“signal detected” or “signal not detected” [defined as the presence or absence of cancer-associated DNA methylation patterns, respectively]) will be returned to participants and their physicians. Participants with a “signal detected” test result will undergo further diagnostic evaluation until diagnostic resolution is achieved. Participants with a “signal not detected” test result will be advised to continue usual medical care and guideline-recommended screening. Cancer status will be assessed in all participants 12 months after enrollment. Participants will also answer questionnaires several times during the study. Cancer risk factors assessed were lifetime smoking history of at least 100 cigarettes, genetic cancer disposition, and history of invasive or hematologic malignancy with definitive treatment 3 or more years before enrollment. ^a^ Participants “with additional risk” had ≥1 of the following risk criteria: a history of smoking (≥100 lifetime cigarettes), personal history of invasive cancer or hematologic malignancy with definitive treatment completed >3 years prior, or be at risk for and/or have a germline mutation associated with cancer susceptibility. ^b^ The diagnostic resolution is defined as the date when the study team determines to end diagnostic evaluation triggered by a “signal detected” test result.

PATHFINDER, a prospective, longitudinal, interventional, multi-center clinical study, will enroll approximately 6200 participants from up to 15 clinical institutions in the US (Figure 1).

Plasma cfDNA will be extracted from 2 tubes of blood and analyzed as described previously [24]. Briefly, plasma cfDNA (up to 75 ng) was subjected to customized bisulfite conversion reaction prepared as a dual indexed sequencing library and enriched using standard hybridization capture conditions for 150-bp paired-end sequencing on the Illumina NovaSeq. Custom software classifies samples and provides two outputs: one indicating ‘cancer signal detected’ or ‘cancer signal not detected’ and the other to predict the origin of the cancer signal. Test results (cancer “signal detected” or “signal not detected”) will be returned to the study investigator and site study team. Participants will be notified of results within 30 days of blood collection.

Participants with a “signal detected” test result will undergo diagnostic tests as determined by the treating physician informed by standard practice guidelines (e.g., National Comprehensive Cancer Network [28,29,30,31]) until diagnostic resolution is reached. Examples of suggested clinical care pathways following a “signal detected” test result for various CSO predictions are shown in Table 1. Participants with a “signal not detected” test result will be advised to continue usual medical care and guideline-recommended screening. The healthcare provider and/or study team, including the principal investigator, sub-investigators, and other study personnel (excluding the study sponsor), will determine the number and types of diagnostic tests to perform and when to end diagnostic evaluation. The treating physician will be responsible for all diagnostic decisions; there are no protocol-required diagnostic procedures. The diagnostic resolution will be defined as the date when the study team (and/or the healthcare provider) ends diagnostic evaluation triggered by a “signal detected” test result. Two diagnostic outcomes will be possible: (1) cancer diagnosis (defined as pathologic or radiologic confirmation of an invasive or hematologic malignancy) or (2) no cancer diagnosis. An additional blood draw may be collected from participants with a “signal detected” test result for research purposes, laboratory process improvements, and proficiency testing.

The expected duration from consent to end of study (EOS) for any study participant will be up to 14 months. While it is expected that most participants will achieve diagnostic resolution during the study period, it is possible that some participants will not.

Clinical information will be collected for all participants from medical records, questionnaires, and MCED test requisition forms. Participants will complete questionnaires before the blood draw, upon return of results, diagnostic resolution, and after 12 ± 1 months from the date of enrollment to assess PROs and perceptions about the MCED test and impact of test results on attitudes towards guideline-recommended screening and subsequent MCED testing. Questionnaires will include the SF-12v2 Health Survey [32], the Adapted Multidimensional Impact of Cancer Risk Assessment [33], and the Patient-Reported Outcome Measurement Information System anxiety 4-item short form [34]. De novo instruments will also be developed to measure participants’ satisfaction with the MCED test and attitudes towards guideline-recommended screening and subsequent MCED tests. A random subset of 50 participants will be selected for a phone interview to assess their comprehension of study communications and educational materials.

All participants will be followed through a medical record review for 12 ± 1 months from the date of enrollment; cancer status will be assessed at Month 12. All study-related adverse events will be documented and reported. No scheduled monitoring of adverse events is planned. The safety of the study will be overseen by the study investigators, study sponsors, reviewing institutional review boards (IRBs), FDA, and a Data Safety Monitoring Board (DSMB). The DSMB will monitor the safety of study interventions, evaluate participant recruitment and accrual, review the study conduct, and provide recommendations to the study sponsor on whether to continue, modify, or stop the study. No formal evaluation of MCED test performance at DSMB review meetings is planned. This study will be conducted in accordance with the protocol, the Declaration of Helsinki and Council for International Organizations of Medical Sciences International Ethical Guidelines, and applicable provisions and conditions of the IRB/IEC, FDA, ICH/Good Clinical Practice Guidelines, and other applicable laws and regulations. The study will be conducted within a total of 7 health networks as follows: Cleveland Clinic (Cleveland, OH; 1 site), Dana-Farber Cancer Institute (Boston, MA; 19 sites); Intermountain (Murray, UT; 3 sites), Mayo Clinic (Rochester, MN; 1 site), Oregon Health Science University (Portland, OR; 4 sites), Sutter (Walnut Creek, CA; 9 sites) and U.S. Oncology (The Woodlands, TX; 19 sites).

### 2.2. Study Objectives and Endpoints

The primary objective will be to assess the extent of diagnostic testing, as determined by the number and types of tests or procedures performed and the time required to achieve diagnostic resolution following a “signal detected” result from the MCED test.

PATHFINDER will have two secondary objectives. The first secondary objective will be to evaluate MCED test performance. The endpoint for this objective will be cancer incidence, assessed through test performance measures for cancer detection (resolution per MCED test, specificity, positive predictive value (PPV), and negative predictive value), and CSO identification (return rate, CSO prediction accuracy) defined later in the text. Sensitivity will not be assessed because it may be subject to substantial bias due to differential verification because only “signal detected” cases are worked up. Cancers included will be invasive solid cancer (stage I–IV, excluding non-metastatic basal cell carcinoma and squamous cell carcinoma of the skin), invasive brain cancer, and hematologic malignancies (including lymphoma (stage I–IV), lymphoid leukemia (without expected staging by the American Joint Committee on Cancer), plasma cell neoplasm (stage I–III), myeloid neoplasms (including myelodysplastic and myeloproliferative neoplasms with behavior code 3 based on ICD-O-3)). The second secondary objective will be to assess PROs and perceptions about the MCED test. Endpoints will include perceptions (e.g., distress, uncertainty, and experience) of MCED test results, health-related quality of life (HRQoL), anxiety, and satisfaction with the MCED test.

PATHFINDER will have six exploratory objectives:

1. Assess participants’ attitudes towards adherence to screening and subsequent MCED testing. Endpoints will include (a) change in attitude towards adherence to guideline-recommended screening [1,2,3,4,5] after an MCED test for participants with “signal not detected” result, and (b) attitude towards subsequent MCED testing.
2. Assess turnaround time for test results from blood draw to when the patient receives results.
3. Evaluate the impact of the COVID-19 pandemic on study endpoints, such as time to diagnostic resolution and participant anxiety levels.
4. Assess the performance of follow-up MCED tests (number and proportion of participants who undergo 1 or more follow-up tests, number and proportion of “signal detected” results, and cancer incidence), health resource utilization, and time needed to achieve diagnostic resolution (e.g., the number of clinic or lab visits, imaging or invasive tests) after a follow-up “signal detected” result.
5. Assess participants’ comprehension of study communications and educational materials about the MCED test.
6. Evaluate changes from baseline in blood biomarkers from multiple blood draws in participants with “signal detected” results.

### 2.3. Eligibility

Participants will be enrolled into two cohorts, depending on cancer risk (Figure 1). Cohort A will include participants who are 50 years or older with at least one of the following additional risk factors: lifetime smoking history of at least 100 cigarettes, genetic cancer disposition, or history of invasive or hematologic malignancy with definitive treatment 3 or more years before enrollment. Cohort B will include participants who are 50 years or older without any of the additional risk factors in Cohort A. Participants with clinical suspicion or diagnosis of cancer or cancer treatment within 3 years of enrollment date will not be eligible for enrollment. Participants with comorbid conditions (e.g., diabetes, hypertension, or coronary artery disease) will not be excluded.

### 2.4. Statistical Analyses

Distribution of the number of clinic or lab visits, imaging or invasive tests, and time to diagnostic resolution will be summarized using descriptive statistics across all participants by the outcome of diagnostic resolution and age group. For participants who will not achieve diagnostic resolution, time to diagnostic resolution will be censored at the time of last contact, electronic medical record (EMR) finding, or EOS. Kaplan–Meier analysis will be used to estimate the probability of achieving diagnostic resolution over time.

Test performance for cancer detection will be evaluated using several performance measures. Resolution per MCED test will be defined as the number and percentage of participants with (1) “signal detected” results and cancer diagnosis at the diagnostic resolution, (2) “signal detected” results and no cancer diagnosis at the diagnostic resolution, (3) “signal detected” results and the diagnostic resolution not achieved, or (4) “signal not detected” results. Overall PPV for cancer detection will be calculated using (1) the proportion of participants with a cancer diagnosis at diagnostic resolution among those who achieved resolution, (2) proportion of participants with a cancer diagnosis at diagnostic resolution among all “signal detected” participants, and (3) proportion of participants with a cancer diagnosis by EOS among all “signal detected” participants. PPV for individual CSO prediction will be assessed as sample size allows. Specificity and NPV will be calculated based on cancer status assessment at EOS. CSO identification will be assessed using return rate and overall CSO prediction accuracy. The return rate will be defined as the proportion of participants with “signal detected” results and determinate CSO returned out of all participants with “signal detected” results with and without cancer diagnosis at diagnostic resolution. The overall accuracy of the most likely CSO prediction will be calculated as the proportion of first CSO predictions that are accurate among participants with returned CSO and cancer diagnosis at resolution and at EOS. Predicted versus diagnosed CSO cancer labels will be compared using a confusion matrix. The accuracy of the second CSO prediction and a combination of the first and second predictions will also be evaluated. Participant characteristics among true positives, false positives, and false negatives will be described.

For PROs and perceptions about the MCED test, questionnaire results will be summarized using descriptive statistics. The distribution of changes in HRQoL and anxiety from baseline to post-baseline assessment at each time point will be summarized. A two-sided t-test will be used to determine if the mean change from baseline differs significantly from zero for each post-baseline assessment.

Descriptive statistics will be used to summarize the following: distribution of time from blood draw to return of result in the analyzable population, responses of all interviewed participants, and changes in blood biomarkers from baseline in participants with research blood draws.

For participants with a “signal not detected” test result, attitude towards adherence to guideline-recommended screening post-test and at EOS will be compared with pre-test attitude.

## 3. Simulated Results

Microsimulations were used to evaluate the expected number of participants with “signal detected” test results and the number of cancers diagnosed at the diagnostic resolution by modeling the natural history of cancer for a set of participants aged 50 and older with and without a history of cancer. To simulate expected results, we estimated PPV because it takes into consideration sensitivity, specificity, and prevalence and is the most clinically meaningful metric physicians use to guide decisions to follow up positive test results. Cancer incidence was estimated to be approximately 1.41% based on data from the Surveillance, Epidemiology, and End Results Program (SEER) database [35], National Institutes of Health–American Association of Retired Persons cohort for current and former smokers [36], and National Health Interview Survey (2001–2014) for current and former smokers [37]. Because the natural history of cfDNA-detectable cancer cases is not established, we explored a range of plausible assumptions for the distribution of dwell times (i.e., how much time each cancer spends in a given stage) per cancer per stage [38]. The test performance was estimated based on results from the second substudy of CCGA (overall sensitivity 55% across > 50 cancer types with a single, fixed false positive rate of <1%) [24,39]. It was assumed that up to 10% of participants might not be analyzable due to clinical or assay evaluability criteria.

Under the most conservative dwell time scenario, which is expected to result in the fewest number of cancers in preclinical states, the median (95% confidence interval [CI]) “signal detected” test results were estimated to be 106 (87–128) among analyzable participants. The number of cancers detected through diagnostic evaluation following a “signal detected” test result was estimated to be 52 (95% CI, 39–67). Thus, overall PPV for cancer detection was estimated to be 49% (95% CI, 39%–58%).

## 4. Discussion

Although several novel cfDNA-based multi-cancer detection strategies have demonstrated the analytical performance needed for population-scale early cancer detection [20,24,40,41], assessment of clinical implementation is limited. The feasibility and safety of MCED tests to be integrated into existing cancer diagnostic workflows and to complement existing guideline-recommended cancer screening tests has been addressed in only one prior study, DETECT-A [22]. In this study, 9911 women received the multi-analyte CancerSEEK test at baseline. In 490 women with abnormal results, an independent test for the abnormal biomarker responsible for the CancerSEEK test result was performed to confirm the result as well as white blood cell (WBC) DNA sequencing to rule out clonal hematopoiesis of indeterminate potential (CHIP). A Multidisciplinary Review Committee reviewed the medical history of 134 women with confirmed positive CancerSEEK results not ruled out by non-cancer-related causes and recommended 127 to undergo full-body positron emission tomography-computed tomography (PET-CT) for further confirmation and tumor localization. Findings from DETECT-A demonstrated that a blood-based multi-cancer test accompanied by PET-CT can be safely integrated into existing cancer diagnostic pathways without affecting adherence to guideline-recommended mammography screening. Newer generations of the CancerSEEK test do not require a confirmatory test to rule out mutations due to CHIP [22].

Final study results are not yet available for PATHFINDER; however, the PATHFINDER study design allows for an initial characterization of clinical use. PATHFINDER will assess the ability of an MCED test to prospectively detect cancer and will shed light on diagnostic yield (i.e., the absolute number of cancers detected) and efforts required to obtain pathologic confirmation of invasive cancer. Notably, the MCED test for PATHFINDER does not require WBC sequencing, uses a locked and validated assay, and predicts CSO to direct the confirmatory diagnostic workup. PATHFINDER will describe the diagnostic pathway for all patients with a “signal detected” result. This will help determine the number of tests and procedures and the amount of time required to work up each positive MCED test to achieve diagnostic resolution. Importantly, PATHFINDER does not mandate any specific diagnostic procedure and supports the clinician’s ability to use their judgment to perform diagnostic workups that are tailored to the patient’s risk factors, personal preferences, and CSO prediction. This aspect of the study will clarify how test results affect care pathways and clinical decision-making and inform strategies for broader implementation of the test in the clinical setting.

Strengths of the PATHFINDER study design include its focus on the population eligible for cancer screening, wide age distribution, the inclusion of participants with a prior cancer history, the inclusion of both men and women, and a large cohort size to ensure that study results are generalizable to a broad population. Distributions of demographic and clinical characteristics will be monitored during enrollment to balance the composition of study cohorts.

As is the case with all simulations, actual observed results may not match simulated results reported here (e.g., if cancer incidence is higher or lower than estimated, or if the actual distribution of cancer types differs from expected distribution).

Similar to many clinical trials [42], the COVID-19 pandemic has disrupted participant enrollment and data collection in PATHFINDER. The use of e-consent or mailed consent forms has provided an efficient means to enroll participants while mitigating COVID-19 exposure risks to participants and staff. However, COVID-19 may also impact timing and choice of diagnostic procedures due to deferral of less urgent procedures, social distancing recommendations, and other infection control measures [43]. These factors may result in longer times to reach a diagnostic resolution, thus underestimating the value of the test, and may introduce selection bias of individuals who are willing to come to the clinic during a pandemic, which will be considered when interpreting study results. The impact of these factors is expected to decrease as COVID-19 exposure risks are mitigated.

## 5. Conclusions

In conclusion, PATHFINDER will help inform the integration of a blood-based MCED test into existing clinical workflows and identify factors that could promote or deter participation in blood-based cancer screening. If successful, the MCED test could help increase the uptake of cancer screening programs, particularly in underserved populations [8,9].

## Figures and Tables

**Table 1:** Proposed clinical care pathways following a “Signal Detected” test result and cancer signal origin prediction.

Cancer Signal Origin Prediction	Proposed First-Line Procedures	
Multiple myeloma	Blood workup including peripheral blood smear, complete blood count (CBC) with differential; chemistry tests including creatinine clearance, protein electrophoresis of blood/urine	
Upper GI (esophagus, stomach)	Blood work	Endoscopy
Colorectal		Colonoscopy
Head and neck		Physical exam, fiber optic exam, ultrasound
Pancreas, gallbladder		CT abdomen with IV contrast, MRCP
Ovary		Abdominal/pelvic exam, ultrasound (preferred)
Lung		CT chest with or without IV contrast
Liver, bile duct		Ultrasound
Breast		Diagnostic mammography with ultrasound (MRI if mammography screening within last 3 months)
Lymphoid neoplasm		CT (neck, chest, abdomen, pelvis) with IV contrast, PET-CT
Indeterminate		CT (neck, chest, abdomen, pelvis) with IV contrast, PET-CT

CT, computed tomography; GI, gastrointestinal; IV, intravenous; MRCP, magnetic resonance cholangiopancreatography; MRI, magnetic resonance imaging; PET-CT, positron emission tomography-computed tomography.
